# Sirenomelia associated with an anterior abdominal wall defect: a case report

**DOI:** 10.1186/s13256-019-2162-0

**Published:** 2019-07-13

**Authors:** Eric Kambale Kavunga, Gabriel Kambale Bunduki, Mupenzi Mumbere, Claude Kasereka Masumbuko

**Affiliations:** 1 Centre de Santé de Référence de Kipese, Nord-Kivu, Democratic Republic of the Congo; 2 Department of Infectious Diseases, Faculty of Medicine, Université Catholique du Graben, PO Box 29, Butembo/Nord-Kivu, Democratic Republic of the Congo; 3 Department of Paediatrics, Cliniques Universitaires du Graben, Faculty of Medicine, Université Catholique du Graben, Butembo, Democratic Republic of the Congo; 4 Department of Surgery, Cliniques Universitaires du Graben, Faculty of Medicine, Université Catholique du Graben, Butembo, Democratic Republic of the Congo

**Keywords:** Sirenomelia, Mermaid syndrome, Abdominal wall defect, Case report

## Abstract

**Background:**

Sirenomelia is a rare and fatal congenital defect. The rarity of this case and its association with abdominal total wall defect drove us to report this case.

**Case presentation:**

We report a rare case of sirenomelia characterized by lower limb fusion, thoracolumbar spinal anomalies, sacrococcygeal agenesis with a rudimentary tail, and genitourinary and anorectal atresia. Coexistent anterior abdominal wall defect in this case highlights its fatalness because of complications associated with the malformation.

**Conclusions:**

Sirenomelia syndrome has seldom been reported. The present case highlights the rare atypical association of sirenomelia with anterior abdominal wall defect. Because the investigations were done in a low-resource setting, the etiology regarding this case remains unclear.

## Background

Sirenomelia, also known as *mermaid syndrome*, is a rare and fatal multisystemic human malformation characterized by malformation of lower limbs as complete or partial fusion of lower limbs into a single lower limb, giving the appearance of a mermaid’s tail [1, 2]. It can be combined with variable visceral abnormalities incompatible with life, most commonly urogenital and gastrointestinal [1–3]; yet, there are a number of reported cases of survival [4, 5]. Up to now, the etiologies of this malformation remain unknown, even if animal experiments in murine models have suggested a genetic basis [1]. The exact incidence of sirenomelia is unknown, but it occurs in 1:60,000 to 1:100,000 births [6]. In the African context, such mermaid-like babies are called “mammy-water babies,” which carries an evil connotation associated with witchcraft.

To our knowledge, we report the first documented case of sirenomelia associated with anterior abdominal wall defect in the Democratic Republic of the Congo.

## Case presentation

Our patient was a 40-year-old Congolese woman married to a nonconsanguineous 43-year-old man. She was of low socioeconomic status, had an unsupervised pregnancy, and her fetus had an unknown gestational age because her last menstrual period was also unknown (she felt pregnant during the lactational amenorrhea). She came to consult for absence of fetal movements for 2 days. She declared that fetal movements were rare during the whole course of the pregnancy (one low-intensity movement per day). She was gravida 11 (G11P10L9D1) and had a previous history of full-term spontaneous vaginal delivery. She had a deceased infant who was issued from her third pregnancy and died in the sixth month of life with febrile gastroenteritis. She noted that one of her children has polydactyly. Her other children are apparently healthy and present no obvious congenital malformations. The patient declared that she took unknown tablet drugs against malaria, which she received from an open market drugstore, during the first term of her pregnancy. She occasionally drinks traditional alcohol (made with fermented maize). She does not smoke or take traditional drugs. She has no history of diabetes in her family. She had no antenatal ultrasonography report, nor were any blood investigations performed.

On clinical examination, the patient was anxious but hemodynamically stable (arterial pressure 120/60 mmHg), afebrile (temperature 36.5 °C), and had a symphysis-fundal height of 31 cm. Fetal heartbeats were absent, and the fetus was in breech presentation. Ultrasonography was performed and revealed a unique fetus with no heartbeat and no movement. Its skull was not perfectly individualized and gave an impression of skull bones overlapping or anencephaly. The femoral length was 54.4 mm (pregnancy age estimated to be 30 weeks) with the fetus in breech presentation. The placenta and genital organs were not visualized. Oligohydramnios was seen with a viscous aspect, giving the impression of maceration. Hence, a cesarean section was indicated for a breech presentation of a macerated intrauterine dead fetus. Intraoperatively, we observed an intrauterine dead fetus in breech presentation, macerated at second degree with polymalformation. The amniotic fluid was green-blackish, and the placenta was friable and weighed 200 g. The fetus weighed 1200 g, had a length of 35 cm, and had a cranial perimeter of 25 cm.

**Fig. 1:**
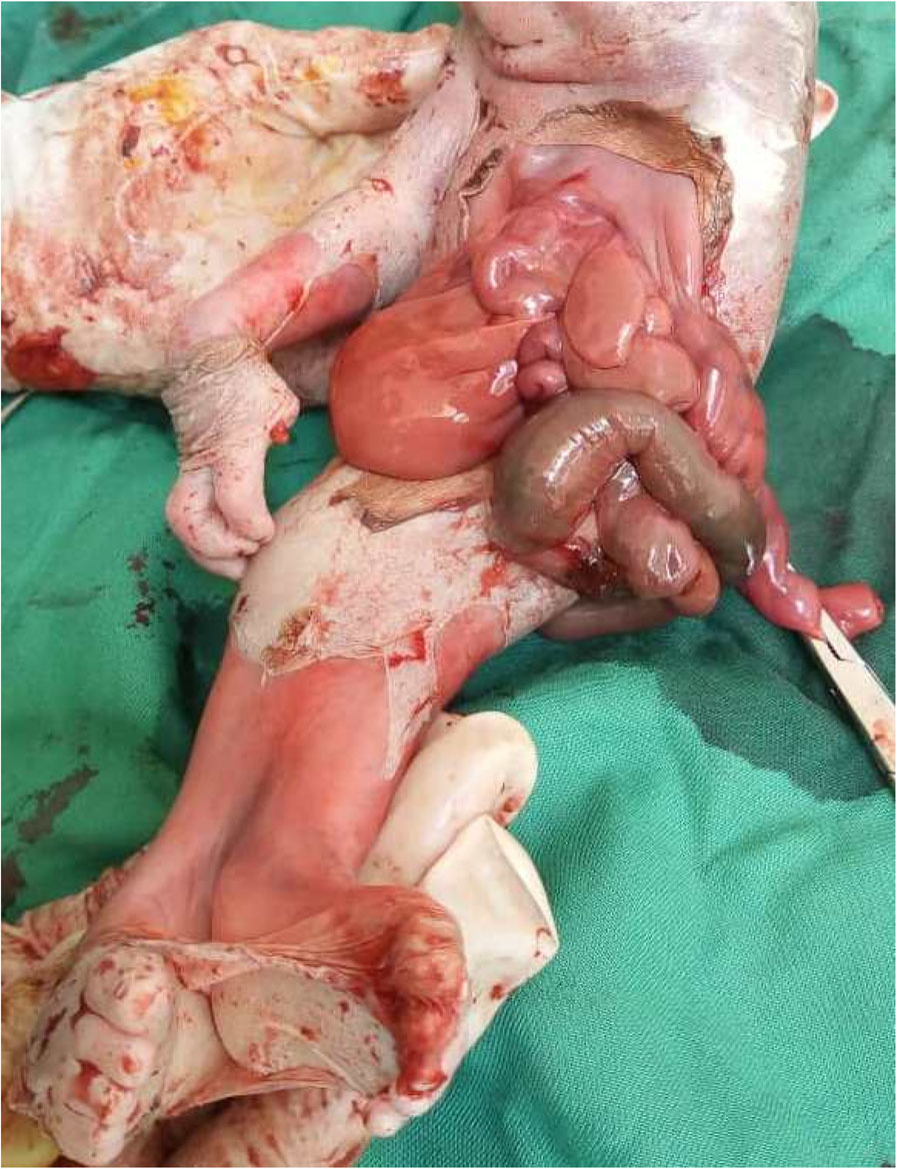
Anterior view of the newborn

**Fig. 2:**
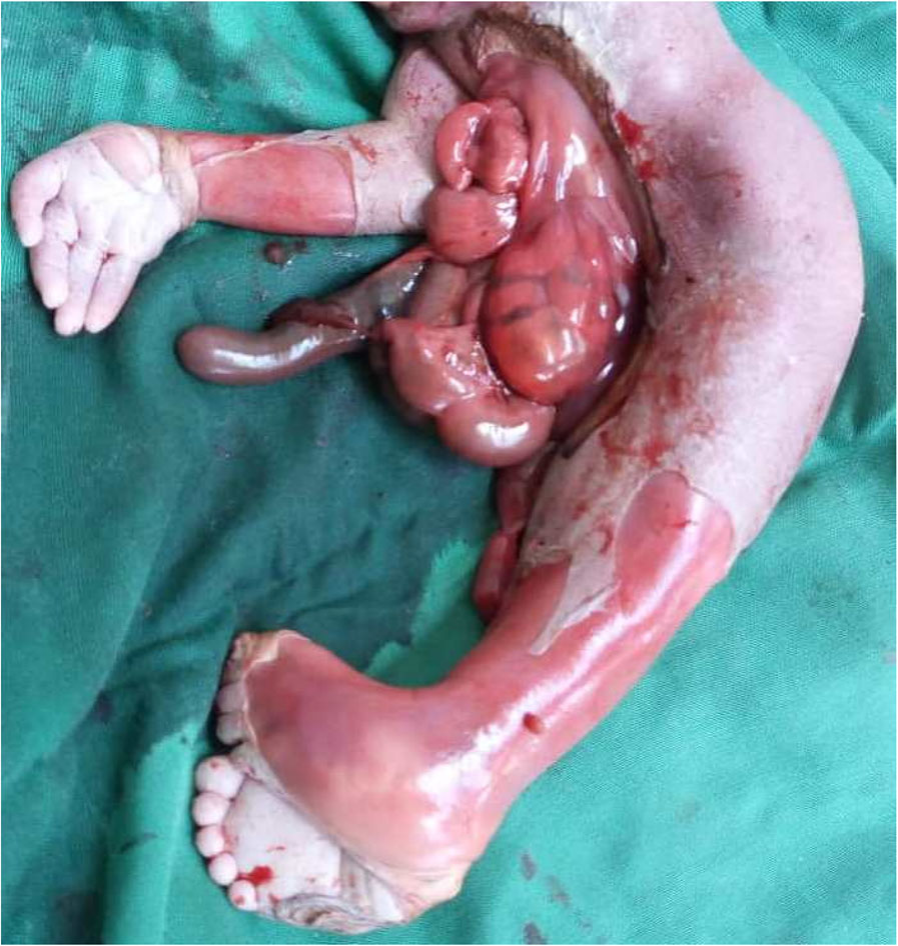
Profile view of the newborn

**Fig. 3:**
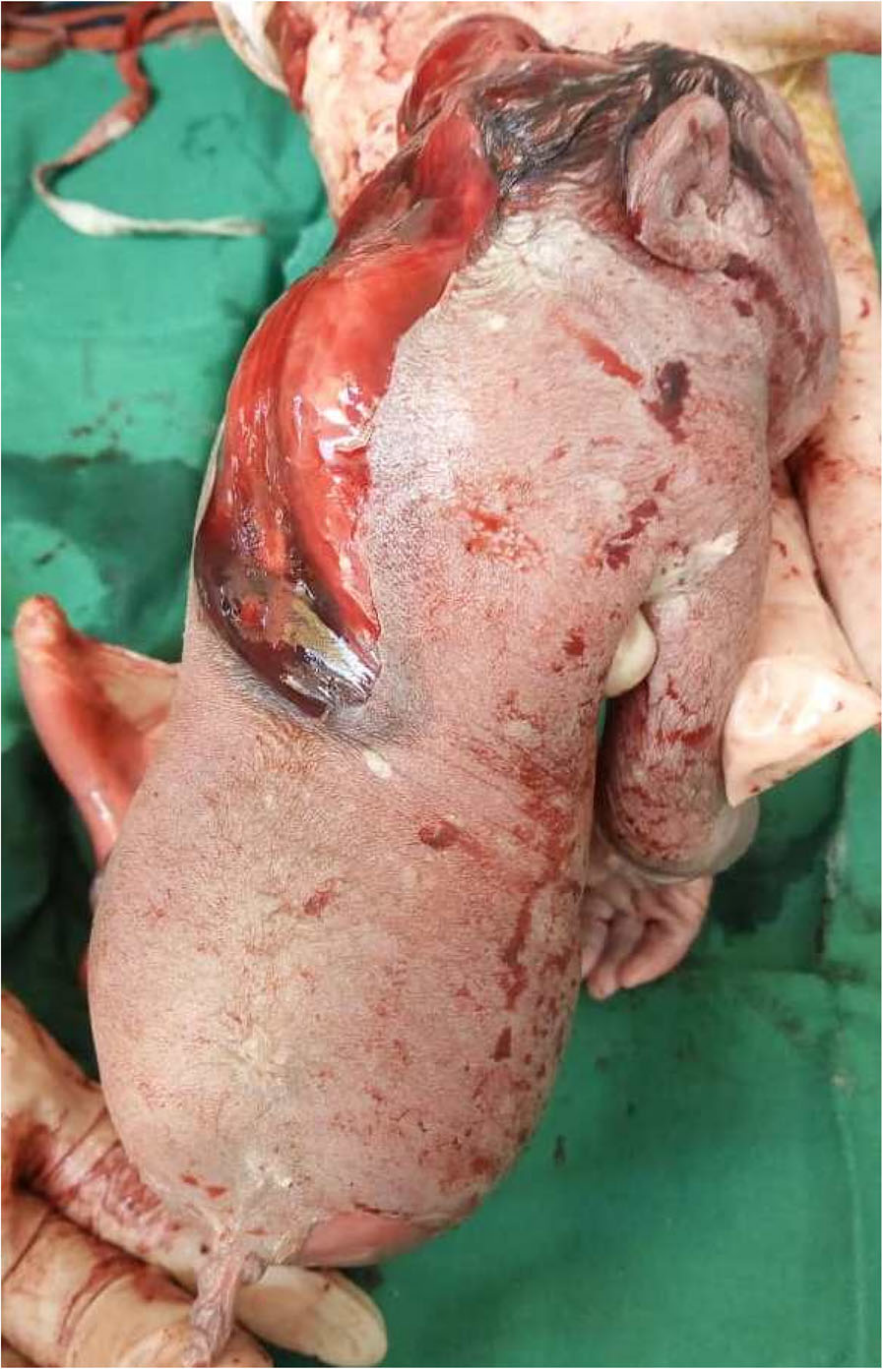
Posterior view of the newborn

On the anterior and profile views of the fetus (Figs. 1 and 2), we observed the following morphological abnormalities: anencephaly, ocular hypertelorism, low-set ears, prominent infraorbital folds, downward-curved nose, and receding chin suggestive of Potter facies; amelia of the left upper limb with the trunk directly attached to the head; agenesia of the anterior abdominal wall with the umbilical cord inserted to something that looked like the omentum; presence of one umbilical artery on the umbilical stump; renal dysgenesis; blind-end colon; undetermined sex (no external genital organs); and absence of urinary meatus. The lower limbs were fused in one single limb from the pelvis, with two feet fused posteriorly, giving two flipper-like feet with five toes on each foot spreading out in a fanlike pattern (ectopode mermaid-like). The external palpation of lower limbs gave the impression of probably two femurs and two tibias. Due to financial constraints, an x-ray was not done. In a posterior view (Fig. 3), we observed a fleshy structure with the appearance of a small, 2-cm tail.

We could not get consent for autopsy or additional explorations on a dead baby, owing to respect for traditional and cultural beliefs. No genetic testing was done due to financial constraints and lack of a genetic diagnostic laboratory in the Democratic Republic of Congo. The mother received 2 g of ampicillin intraoperatively and underwent a bilateral tubal ligation. She was maintained in the hospital for observation. She received adequate postpartum counseling and was discharged on day 7 postpartum.

## Discussion and conclusions

Sirenomelia is rare and is usually fatal within a day or two of birth because of complications related to abnormal kidney, lung, heart, and bladder development and function [7]. Our case was a macerated intrauterine dead fetus. The case is rare, and many healthcare professionals might not have come across a case of mermaid syndrome in their entire professional practice.

The etiology and pathogenesis of this malformation is undetermined. Most cases occur randomly for nonapparent reasons [1], as in the case we report. However, maternal diabetes mellitus [8], genetic predisposition, environmental factors (tobacco use, retinoic acid and heavy metal exposure), and vascular steal phenomenon with the single vitelline umbilical artery diverting blood supply and nutrients from the lower body and limbs [9–11] have been reported as possible etiological factors. In our patient’s case, there was no history of maternal diabetes and no history of tobacco use before and during pregnancy.

Although the genetic predisposition has been raised, etiology of sirenomelia remains unclear. There is no report on instances of familial recurrence of sirenomelia [12, 13]. The results of karyotype testing done on sirenomelia cases reported in the literature are almost all normal. Meanwhile, a recent report showed a case of sirenomelia in a fetus with triploid mosaic (69,XXX/46,XX) [14]. Another case of sirenomelia with a reciprocal translocation 46X,t(X;16)(p11.23;p12.3) has also been reported; however, the chromosomal breakpoints on the pairs of chromosomes did not disrupt the coding genes associated with early human development, especially with blastogenesis [15]. In murine models with sirenomelia, mutations in the superfamily of cytochrome P450 (CYP) genes, specifically CYP26A1, an enzyme that degrades retinoic acid, have been reported [1]. Another observation is the link between bone morphogenetic protein 7 (BMP7) and twisted gastrulation (Tsg); loss of BMP7 combined with a complete loss or half-dose of Tsg in murine models was associated with sirenomelia [16].

None of these studies have been replicated in human models. Therefore, the molecular mechanisms producing sirenomelia remain unclear, even if two pathophysiological hypotheses have been proposed as explanations: the vascular steal theory [10] and defective blastogenesis or failure of the development of ventral mesoderm [17]. These two pathophysiological hypotheses could be interrelated and may constitute a similar pathophysiological continuum. Abnormalities of blastogenesis would result in defects of the caudal vasculature of the embryo, leading to malformation of the targeted organs by ischemia and nutrient deficiency [18].

The pattern of birth defects seen in sirenomelia is associated with abnormal umbilical cord blood vessels. Most babies with sirenomelia have only one umbilical artery and one vein [19], as was seen in our patient’s case.

Multiple abnormalities highlighted in our patient’s case showed a complete anterior abdominal wall agenesis with most of the exposed viscera, fusion of lower limbs, fusion of the lower spine, Potter syndrome, left upper limb amelia, and anencephalia. None of the cases described in the literature has described the severity of the highlighted clinical features in our case report. Only a few rare noteworthy papers have reported sirenomelia associated with abdominal wall defect [12]. The possible pathophysiology of abdominal wall defect in sirenomelia may be the vascular steal theory [10] and failure of the development of ventral mesoderm [17]. In fact, the single artery present (steal vessel) diverts the flow of blood that normally circulates from the aorta to the lower parts of the embryo and to the placenta. Thus, the steal vessel redirects the blood flow to the placenta without ever reaching the tail end (caudal) of the embryo. As a result of this rerouted blood flow, the steal vessel also diverts nutrients away from the blood-deprived portion of the embryo. Arteries in this caudal area are underdeveloped, and tissues dependent on them for nutrient supply fail to develop, are malformed, or arrest their growth in some incomplete stage. The ventral mesoderm may also be affected with this phenomenon; thus, a failure in its development causes an abdominal wall defect.

An association between caudal regression syndrome, VACTERL association (vertebral defects, anal atresia, cardiac defects, tracheoesophageal fistula, renal anomalies, and limb abnormalities), and sirenomelia has been reported [20–22]. Single umbilical artery and renal anomalies are almost invariably present, whereas gastrointestinal anomalies are variable and include a blind-ending colon, rectal atresia, and anal imperforation. Vertebral defects, cardiac defects, esophageal atresia with tracheoesophageal fistula, radial agenesis, upper limb defects, and anomalies of the central nervous system can also be found with sirenomelia. Although we did not perform an autopsy, some of the associated anomalies in the VACTERL spectrum were present: single umbilical artery, anogenital anomalies, anencephalia, sacrococcygeal agenesis, and upper limb defects. The clinical phenotypic overlap between caudal dysgenesis, VACTERL association, and sirenomelia in our patients is highlighted, lending support to the theory that these entities may be different manifestations of a single pathogenic process [20, 21].

Although we did not have x-rays to categorize our case with certitude, on the basis of external examination, we infer that our case met criteria for type III (all thigh and leg bones present except the fibula) of the Stocker and Heifetz classification [23].

In the literature, few cases are reported worldwide, among which rare cases are from Africa [19]. There are approximately 300 cases reported in the literature, 15% of which are associated with twinning, most often monozygotic. In the antenatal period, sirenomelia can be diagnosed as early as 13 weeks by using high-resolution or color Doppler sonography [7]. In our case, the diagnosis was made after birth due to our resource-limited context and involved only one fetus.

Sirenomelia is a very rare fatal congenital malformation. To our knowledge, this is the first case reported from Central Africa in a set association with an abdominal wall defect. This report adds to existing knowledge and data about this condition. The resource-poor setting in which we are working and the respect for traditional and cultural beliefs limited the investigations and description of the full spectrum of sirenomelia in this reported case.

## Data Availability

The datasets used and/or analyzed during the current study are available from the corresponding author on reasonable request.

## Abbreviations

BMP7: Bone morphogenetic protein 7
Tsg: Twisted gastrulation
